# Supplementary material for: Development and Characterization of Bioactive Polypropylene Films for Food Packaging Applications
Source: Polymers (Basel). 2021 Oct 11;13(20):3478. doi: 10.3390/polym13203478 (PMC8538041; doi:10.3390/polym13203478)
Supplement: Supplementary file 1 [file polymers-13-03478-s001.zip › polymers-1326450-supplementary.pdf]

polymers

MDPI

Supplementary Materials

Table S1. Parameters of the PP regranulation processor

Heating Zones		Temperature [°C]	
		Modified pellet mono	*Modified pellet mix*
Extruder Zone	***1***	170	170
	2	175	180
	3	175	180
	4	175	180
	5	175	180
	6	180	180
	7	180	180
	8	180	180
	9	185	180
	10	185	185
Die		190	185

Table S2. Cast processing parameters of modified PP film extrusion

Heating Zones		Temperature [°C]		
		Granules	*Modified pellet mono*	*Modified pellet mix*
Extruder Zone	***1***	175	175	160
	2	180	180	165
	3	185	185	175
	4	195	185	185
Pipe		200	185	180
CoEx	***1***	205	190	180
	2	205	190	180
Die	***1***	210	195	185
	2	210	195	185
	3	210	195	185

Along with the complexity of premixes - pellet; → mono-modified pellet(they contain a plasticizing additive or a bioactive compound); → modified mix pellet

Table S3. Modifications of PP by plasticizers and bioactive - single additives

**Plasticizer**		**Bioactive Agent**		**Regranulate**	**Cast Extrusion**
Acronym	*Content [%]*	*Acronym*	*Content [%]*		
A121	5	-	-	+	+
CO	5	-	-	+	+
-	-	RE	5	+	+
-	-		7.5	-	-
-	-	OO	5	+	+
-	-		7.5	+	+
-	-		10	+	+
-	-	GTE	5	+	+
-	-		7.5	-	-
-	-	MP	5	-	-

where: + - prepared; - - not prepared

Table S4. Modifications of PP by plasticizers and bioactive - double additives

**Plasticizer**		**Bioactive Agent**		**Regranulate**	**Cast Extrusion**
Acronym	*Content [%]*	*Acronym*	*Content [%]*		
A121	5	RE	2.5	+	+
	5		5	+	+
CO	2.5		2.5	+	+
	2.5		5	+	+
-	-	OO	2.5	+	+
		RE	5		
-	-	OO	5	+	+
		MP	5		
CO	5	GTE	5	+	+
A121	5	GTE	5	+	+

CO	5	RE	5	+	+
-	-	GTE	5	+	+
		OO	5		
-	-	GTE	5	+	+
		OO	5		

where: + - prepared; - - not prepared

Table S5. Modifications of PP by plasticizers and bioactive - triple additives

**Plasticizer**		**Bioactive Agent**		**Regranulate**	**Cast Extrusion**
Acronym	*Content [%]*	*Acronym*	*Content [%]*		
-	-	OO	5	+	+
		MP	2.5		
		RE	2.5		
CO	2.5	OO	5	+	+
		MP	2.5		
-	-	GTE	2.5	+	+
		RE	2.5		
		OO	5		
-	-	GTE	2.5	+	+
		MP	1		
		OO	5		

where: + - prepared; - - not prepared

Table S6. Modifications of PP by plasticizers and bioactive - multi additives

**Plasticizer**		**Bioactive Agent**		**Regranulate**	**Cast Extrusion**
Acronym	*Content [%]*	*Acronym*	*Content [%]*		
CO	1.75	OO	5	+	+
		GTE	2.5		
		MP	1		
-	-	OO	5	+	+
		MP	2.5		
		GTE	2.5		
		RE	2.5		
CO	1.75	OO	5	+	+
		RE	2.5		
		GTE	2.5		
		MP	1		

where: + - prepared; - - not prepared

Table S7. Antimicrobial activity of single additive extruded PP films against bacteria using ASTM E 2180-18 procedure

**Active substance**		*B.subtilis*	*E.coli*	*S.aureus*	*P.putida*
Acronym	*[%]*				
A121	5	No effect	No effect	No effect	No effect
CO	5	No effect	No effect	No effect	No effect
GTE	5	1-1.5 log reduction	No effect	No effect	Total reduction

RE	5	No effect	No effect	No effect	No effect
OO	5	No effect	No effect	No effect	No effect
	7.5	1-1.5 log reduction	No effect	Total reduction	1-1.5 log reduction
	10	No effect	1-1.5 log reduction	Total reduction	No effect

Table S8. Antimicrobial activity of single additive extruded PP films against yeast and molds

**Active substance**		*C.albicans*	*A.alternata*	*A.brasiliensis*	*P.expansum*	*F.oxysporu*
Acronym	*[%]*					
A121	5	No effect	No effect	No effect	No effect	No effect
CO	5	No effect	1-1.5 log reduction	No effect	No effect	No effect
GTE	5	No effect	No effect	No effect	No effect	No effect
RE	5	No effect	1-1.5 log reduction	No effect	No effect	1-1.5 log reduction
OO	5	No effect	1-1.5 log reduction	No effect	No effect	1-1.5 log reduction
	7.5	>2log reduction	1-1.5 log reduction	No effect	No effect	>2log reduction
	10	Total reduction	Total reduction	No effect	No effect	Total reduction

Table S9. Antimicrobial activity of double additives extruded PP films against bacteria using ASTM E 2180-18 procedure.

**PP with Active substance**		*B.subtilis*	*E.coli*	*S.aureus*	*P.putida*
Acronym	*[%]*				
RE	2.5	No effect			
CO	2.5				
RE	5	1-1.5 log reduction	No effect	Total reduction	No effect
CO	2.5				
**RE**	**5**	**>2log reduction**	**>2log reduction**	**Total reduction**	**1-1.5 log reduction**
**OO**	**5**				
OO	5	1-1.5 log reduction	No effect	Total reduction	1-1.5 log reduction
MP	5				
RE	2.5	No effect			
A121	2.5				
RE	5	No effect	No effect	Total reduction	1-1.5 log reduction
A121	5				
**OO**	**5**	**Total reduction**			
**CO**	**5**				
GTE	5	1-1.5 log reduction	No effect	1-1.5 log reduction	Total reduction
CO	5				
GTE	5	1-1.5 log reduction	No effect	Total reduction	Total reduction
A121	5				
GTE	5	1-1.5 log reduction	No effect	Total reduction	Total reduction
OO	5				

Table S10. Results obtained using ASTM E 2180-18 for double additives active substance extruded films against yeast and molds.

**PP with Active substance**		*C.albicans*	*A.alternata*	*A.brasiliensis*	*P.expansum*	*F.oxysporum*
Acronym	*[%]*					
RE	2.5	No effect				1-1.5 log reduction
CO	2.5					
RE	5	No effect				Total reduction
CO	2.5					
**RE**	**5**	**>2log reduction**	**Total reduction**	**No effect**	**No effect**	**1-1.5 log reduction**
**OO**	**5**					
OO	5	>2log reduction	1-1.5 log reduction	No effect	No effect	>2log reduction
MP	5					
RE	2.5	No effect	No effect	No effect	No effect	1-1.5 log reduction
A121	2.5					
RE	5	No effect				
A121	5					
**OO**	**5**	**Total reduction**	**Total reduction**	**1-1.5 log reduction**	**No effect**	**Total reduction**
**CO**	**5**					
GTE	5	No effect				
CO	5					
GTE	5	No effect				
A121	5					
GTE	5	Total reduction	Total reduction	No effect	No effect	>2log reduction
OO	5					

Table S11. Results using ASTM E 2180-18 for different films multi additives mixtures against bacteria.

**PP with Active substance**		*B.subtilis*	*E.coli*	*S.aureus*	*P.putida*
Acronym	*[%]*				
OO	5	No effect	No effect	Total reduction	>2log reduction
RE	2.5				
MP	2.5				
**OO**	**5**	**>2log reduction**	**Total reduction**	**Total reduction**	**>2log reduction**
**RE**	**2.5**				
**CO**	**1.75**				
MP	2.5	No effect	No effect	Total reduction	>2log reduction
OO	5				
GTE	2.5				
GTE	2.5	1-1.5 log reduction	No effect	Total reduction	Total reduction
MP	2.5				
OO	5				
RE	2.5				
**MP**	**1**	**1-1.5 log reduction**		**Total reduction**	
**OO**	**5**				
**RE**	**2.5**				
**CO**	**1.75**				
GTE	2.5	1-1.5 log reduction	No effect	Total reduction	Total reduction
MP	1				
OO	5				
RE	2.5				
CO	1.75				

Table S12. Results using ASTM E 2180-18 for different films multi additives mixtures against yeast and molds.

**PP with Active substance**		*C.albicans*	*A.alternata*	*A.brasiliensis*	*P.expansum*	*F.oxysporum*
Acronym	*[%]*					
OO	5	Total reduction	1-1.5 log reduction	No effect	No effect	1-1.5 log reduction
RE	2.5					
MP	2.5					
**OO**	**5**	**Total reduction**	**Total reduction**	**1-1.5 log reduction**	**1-1.5 log reduction**	**Total reduction**
**RE**	**2.5**					
**CO**	**1.75**					
MP	2.5	Total reduction	1-1.5 log reduction	No effect	No effect	1-1.5 log reduction
OO	5					
GTE	2.5					
GTE	2.5	Total reduction	Total reduction	No effect	No effect	>2log reduction
MP	2.5					
OO	5					
RE	2.5					
**MP**	**1**	**Total reduction**	**Total reduction**	**No effect**	**No effect**	**Total reduction**
**OO**	**5**					
**RE**	**2.5**					
**CO**	**1.75**					
GTE	2,5	Total reduction	Total reduction	No effect	No effect	Total reduction
MP	1					
OO	5					
RE	2.5					
CO	1.75					

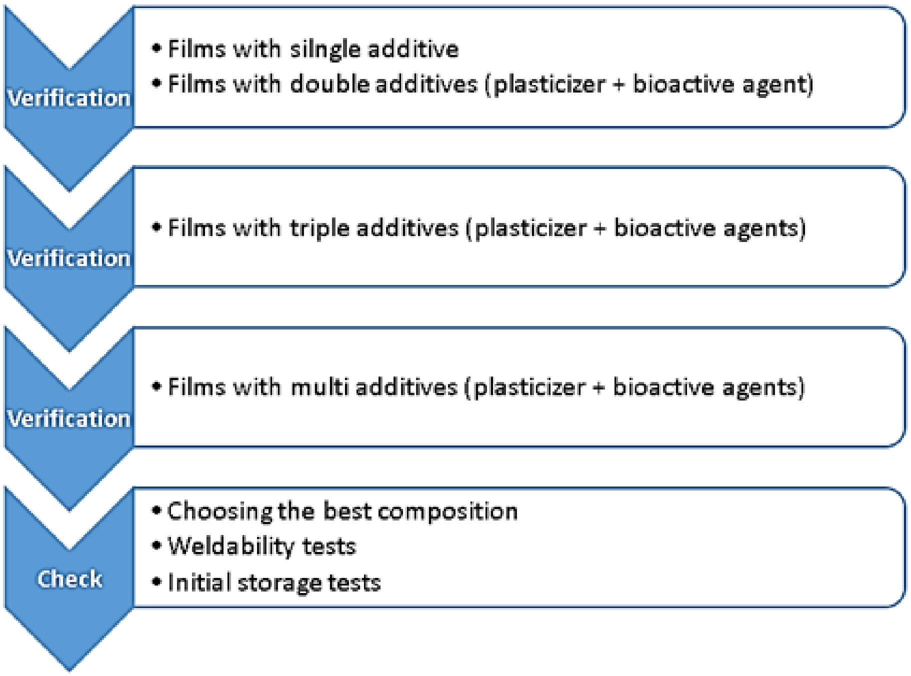

Figure S1. Experimental design
